# The Gadolinium (Gd^3+^) and Tin (Sn^4+^) Co-doped BiFeO_3_ Nanoparticles as New Solar Light Active Photocatalyst

**DOI:** 10.1038/srep42493

**Published:** 2017-02-14

**Authors:** Syed Irfan, Syed Rizwan, Yang Shen, Liangliang Li, A Asfandiyar, Sajid Butt, Ce-Wen Nan

**Affiliations:** 1 State Key Laboratory of New Ceramics and Fine Processing, School of Materials Science and Engineering, Tsinghua University, Beijing 100084, China; 2 Department of Physics, School of Natural Sciences (SNS), National University of Science and Technology (NUST), Islamabad 44000, Pakistan; 3 Department of Materials Science and Engineering, Institute of Space Technology, Islamabad, 44000, Pakistan

## Abstract

The process of photocatalysis is appealing to huge interest motivated by the great promise of addressing current energy and environmental issues through converting solar light directly into chemical energy. However, an efficient solar energy harvesting for photocatalysis remains a critical challenge. Here, we reported a new full solar spectrum driven photocatalyst by co-doping of Gd^3+^ and Sn^4+^ into A and B-sites of BiFeO_3_ simultaneously. The co-doping of Gd^3+^ and Sn^4+^ played a key role in hampering the recombination of electron-hole pairs and shifted the band-gap of BiFeO_3_ from 2.10 eV to 2.03 eV. The Brunauer-Emmett-Teller (BET) measurement confirmed that the co-doping of Gd^3+^ and Sn^4+^ into BiFeO_3_ increased the surface area and porosity, and thus the photocatalytic activity of the Bi_0.90_Gd_0.10_Fe_0.95_Sn_0.05_O_3_ system was significantly improved. Our work proposed a new photocatalyst that could degrade various organic dyes like Congo red, Methylene blue, and Methyl violet under irradiation with different light wavelengths and gave guidance for designing more efficient photocatalysts.

There is an increasing concern over the sudden increase in environmental pollution, especially from industrial waste matter as it has destroyed our aquatic environment. Many researchers are looking for an efficient way to clean the wastewater. The semiconductor-based photocatalysts have become a major research field for removing and degrading the hazardous compounds in water due to their compatibility as an environmental pollution mediator^1^,^2^. The photo-excited nanoparticles can generate electron-hole pairs by absorbing the sun light. These photo-excited electrons and holes are capable to activate the redox reactions among pollutants. Despite the fact that numerous semiconductor systems including TiO_2_, ZnO_2_, and WO_3_ 3,4,5 have been used and are still under examination, the current productivity of photocatalysis in existing systems is not satisfactory. A number of associated factors limit the performance of the photocatalysts, including recombination between photo-excited electrons and holes, low consumption of visible-light because of the mismatch between the semiconductor band-gap and the solar spectrum, material instability in the redox environment, etc. In general, the driving force that directly separates and transports charges is the most significant factor^6^,^7^,^8^.

Recently, the use of ferroelectric materials to convert light into mechanical^9^,^10^, electrical^11^,^12^, or chemical^13^,^14^ energy has generated huge interest for understanding the mechanisms as well as for applications in photovoltaic, photocatalytic, and photo-transducer devices^15^. The great potential for applications comes from their unique ferroelectrics properties and the spontaneous electric polarization due to the breaking of a strong inversion symmetry^16^. Ferroelectrics reveal an intrinsic spontaneous polarization that is at the heart of the aforementioned photo-induced phenomena. Indeed, the light excitation produces electron-hole pairs; the polarization serves as an internal electric field promoting the charge carrier separation. By stimulating the separation of photo-excited carriers to a desirable point, the spontaneous polarization of ferroelectric materials could be used to design new photovoltaic devices; therefore, these materials have been intensively studied for photovoltaic applications^17^,^18^ Ferroelectric materials can also serve as new candidates for photocatalysis with similar advantage and mechanism. Many researchers have found that ferroelectric materials can do much better photocatalysis than other materials. More interestingly, the photo-generated charges can move to the surface of material and act as redox sources for degradation of contaminant molecules in wastewater treatment^13^,^19^. These redox sources can also be used for water splitting in sustainable hydrogen fuel cells^14^,^20^. In recent past, a lot of effort has been done for Bi-based photocatalysts used for photocatalysis purpose in different wavelengths ranging from ultraviolet (UV) to near infrared (NIR) region^21^,^22^,^23^,^24^,^25^.

Recently, BiFeO_3_ (BFO) has attracted a huge attention for photo-induced applications due to its relatively small band-gap (*E*_*g*_ is 2.6–2.8 eV) in comparison with other ferroelectric oxides such as like BaTiO_3_, LiNbO_3_, and PbZrTiO_3_ (*E*_*g*_ > 3 eV), allowing one to utilize a larger part of the sunlight spectrum. In addition, its larger polarization value (*P* ≈ 100 μC/cm^2^) provides a more efficient separation for the photo-generated charge carriers^13^,^26^. Therefore, besides its photovoltaicity^18^, there is an increasing interest for its use in photolysis and photocatalytic activity under visible-light irradiation^13^,^14^,^16^,^17^. BFO could become a substitute to the widely investigated photocatalytic material TiO_2_ that has a larger band-gap^27^. It has been shown that the micrometer-sized BFO particles exhibit efficient photo-absorption due to {111}-cubic like facets^28^,^29^. Core-shell nanostructures based on BFO coated with TiO_2_ have also been used to enhance the surface reactivity^30^. Doping of Bi^3+^ site with rare earth and alkaline earth metal elements and doping of Fe^3+^ site with transition metal elements have been recently studied^31^,^32^,^33^,^34^. The co-substitution at Bi^3+^ and Fe^3+^ in BFO has also been reported^35^,^36^,^37^,^38^,^39^,^40^,^41^,^42^. It has been reported that the doping of Gd^3+^ at A-site onto BFO shows enhanced photocatalytic degradation of Rhodamine B due to ferromagnetic behavior^43^. Additionally, the effects of Sn^4+^ doping on the morphology and electromagnetic properties of BFO have been studied^44^,^45^.

In this report, we simultaneously doped Gd^3+^ and Sn^4+^ into A and B-sites of BFO, respectively. The Bi_1−x_Gd_x_Fe_1−y_Sn_y_O_3_ nanoparticles with different doping concentrations were synthesized with a double solvent sol-gel method. The enhanced photocatalytic properties of BGFSO nanoparticles under UV-*vis*-NIR light were observed and discussed.

## Results and Discussion

**Figure 1:**
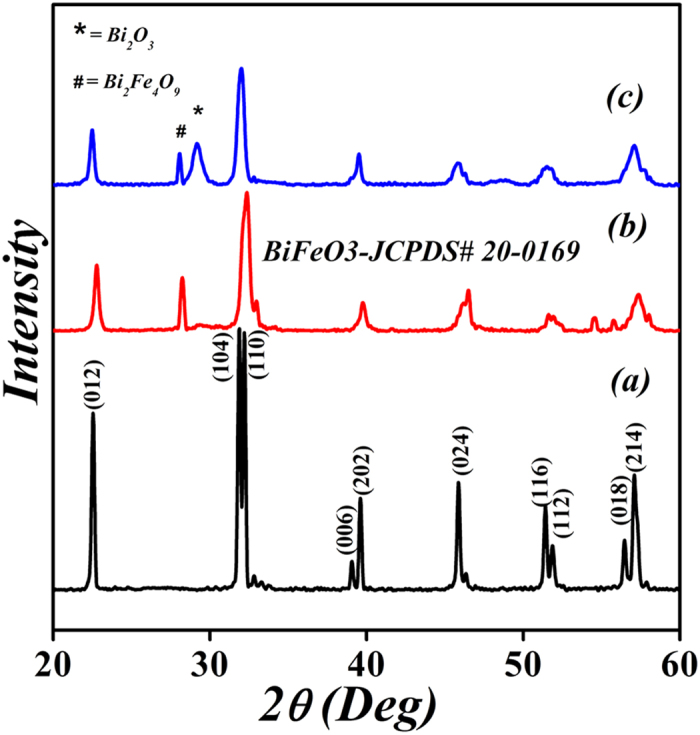
XRD spectra of BGFSO obtained by Gd^3+^ and Sn^4+^ co-doping. (**a**) BFO, (**b**) BGFO-5Sn, and (**c**) BGFO-10Sn.

**Figure 2:**
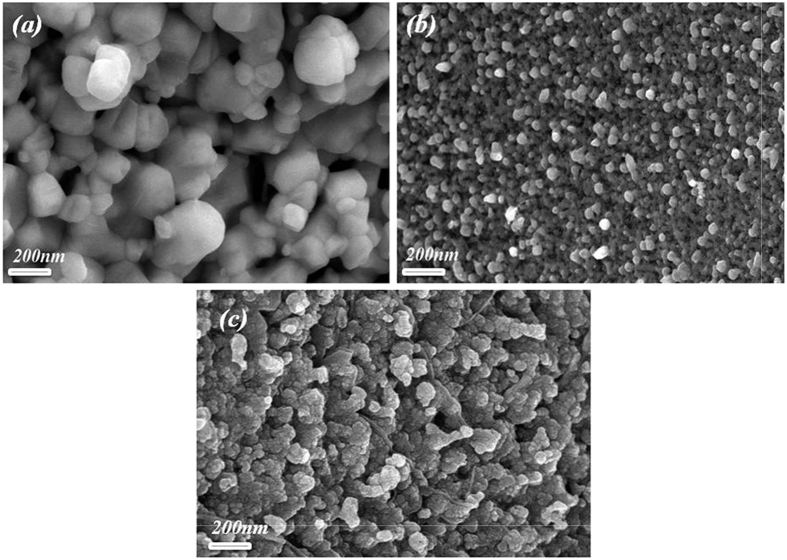
SEM images of (**a**) BFO, (**b**) BGFO-5Sn, and (**c**) BGFO-10Sn. nanoparticles.

**Figure 3:**
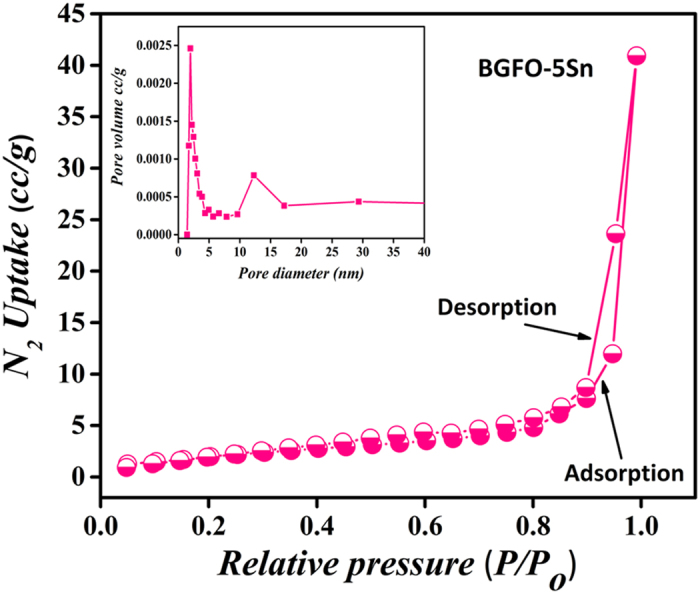
N_2_ gas isotherm measured at 77K for BGFO-5Sn. The inset is the differential pore size distribution curve from BJH method.

Figure 1 shows the X-ray diffraction (XRD) patterns of Gd^3+^ and Sn^4+^ co-doped BFO nanoparticles. The diffraction peaks of doped BFO were identified as a polycrystalline rhombohedrally-distorted perovskite structure with an R3c space group (JCPDS card No. 20–0169) along with the existence of minority phases such as Bi_2_Fe_4_O_9_ and Bi_2_O_3_. The impact of Gd^3+^ and Sn^4+^ doping on the structure can be clearly seen as the (104) and (110) doublet diffraction peaks of Gd^3+^ and Sn^4+^ doped BFO near 2θ ∼ 32° become a single sharp peak that shifts to a lower diffraction angle with increasing amount of Sn^4+^ substitution. This observation indicates an expansion of the unit cell due to the substitution of the Fe^3+^ ion by Sn^4+^ having a larger ionic radius. The grain sizes calculated by using Scherrer’s formula are 60, 18, and 22 nm for BFO, BGFO-5Sn, and BGFO-10Sn, respectively^46^. It is found that the shape of pure BFO nanoparticles is irregular and non-uniform as shown in Fig. 2. The BGFO-5Sn seems more porous compared to the pure BFO nanoparticles suggesting that it might be more suitable for the photocatalytic activity under visible-light irradiation. Figure 3 shows the N_2_ gas isotherm of BGFO-5Sn at 77 K. This is a type IV isotherm, where the initial region is closely related to Type-II isotherm. Type IV isotherm is exhibited by mesoporous adsorbents. The hysteresis is due to the filling and emptying of the mesopores by capillary condensation^47^. The Brunauer-Emmett-Teller (BET) surface area measurements were carried out via multi-point BET method using adsorption calculations in a relative pressure (*P/P*_*o*_) range of 0.05 to 0.25. The pore size distribution was calculated by desorption isotherms using the Barret-Joyner-Halender (BJH) method^48^,^49^. The isotherms of other samples can be seen in Figure S1 (see Supplementary Information). The BGFO-5Sn possesses a surface area of 15.0 m^2^/g, which is evidently low, probably owing to the random orientation of pores in the structure and highly crystalline nature of nanoparticles^50^. Nevertheless, an average pore size of 2.2 nm can be seen from the differential pore size distribution curve in the inset of Fig. 3. The comparison for the BET surface area of all samples is shown in Table S1 (Supplementary Information). The narrow hysteresis and pore size distribution of BGFO-5Sn clearly indicates the presence of mesopores in the structure. Thus, it can be concluded that BGFO-5Sn is a highly crystalline mesoporous material.

### Band Gap Engineering

**Figure 4:**
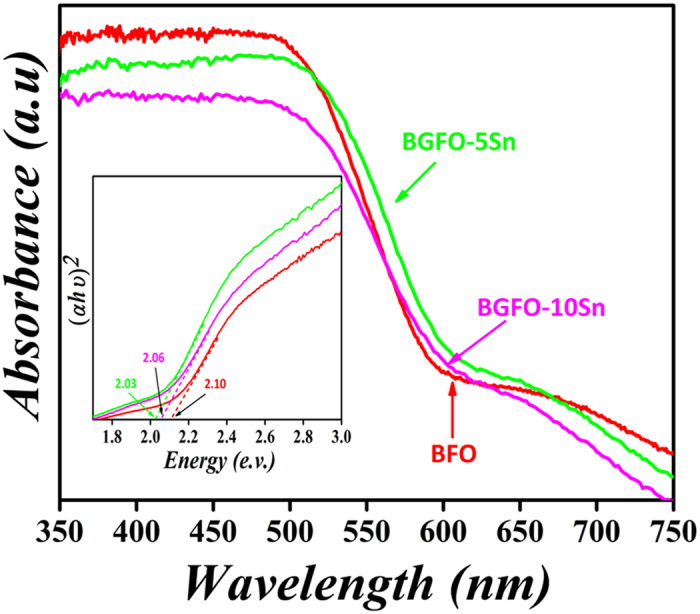
UV-*vis* absorption spectra of BFO and BGFSO. The inset shows the calculation of the corresponding band-gaps.

The UV–vis absorption peaks for BFO, BGFO-5Sn and, BGFO-10Sn are shown in Fig. 4. It is clear that there is a spectrum shift when BFO is substituted with Sn^4+^, which increases the band-gap^51^. The band-gap of BFO first decreases from 2.10 eV to 2.03 eV with doping of Gd^3+^ and Sn^4+^, but then increases up to 2.06 eV with increasing concentration of Sn^4+^ into BGFO; such an increment is supported by the first-principles calculations^52^. This increase in band-gap may result in an improvement of photocatalytic activity in Gd^3+^ and Sn^4+^ co-doped BFO nanoparticles^53^. Our results propose that the band-gap of BFO can be tuned by co-doping of Gd^3+^ and Sn^4+^ to increase its operational range for degradation of organic pollutants.

### Photocatalytic Activity

The photocatalytic activity of as-prepared BGFSO powder samples was observed by photo-degradation of organic dyes such as Congo red (CR), Methylene blue (MB), and Methyl violet (MV) (100 mg/L) aqueous solution. Typically, 0.10 g photo-catalyst powder was dispersed into 100 mL dyes solution and stirred in dark for 2 h to reach the adsorption-desorption equilibrium between the photo-catalyst and organic dye molecules. To avoid thermal effect during the degradation process, ice bath and magnetic stirring were hold continuously to keep the solution uniformity (to get a homogenous solution). A 5 W LED with an emission wavelength of 365 ± 5 nm was used as the UV light source. A 300 W Xenon lamp with 420 nm and 800 nm cut-off filters were used as visible and NIR light sources, respectively. The incident light source was positioned at above the aqueous solution vertically with light intensity of 78 mW/cm^2^, 132 mW/cm^2^, and 473 mW/cm^2^ for UV, visible, and NIR lights, respectively. The 3 mL suspension was collected and centrifuged after every 30 minutes interval and the residual of CR, MB, and MV in the supernatant was investigated by UV-*vis* spectrophotometer. The photocatalytic activity was carried out under the same conditions. Three organic dyes having different chromophores were chosen to study the photocatalytic degradation. Congo red is the sodium salt of benzidinediazo-bis-1-naphthylamine-4-sulfonic acid, Methylene blue is from heteropolyaromatic dye, and triphenylmethane is also known as the Methyl violet. The degradation rate depends on the structure of organic dye, light intensity, pH of the medium, illumination source, dye concentration, and catalyst morphology. The degradation efficiency of organic dyes is determined by using following formula,


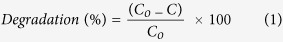


**Figure 5:**
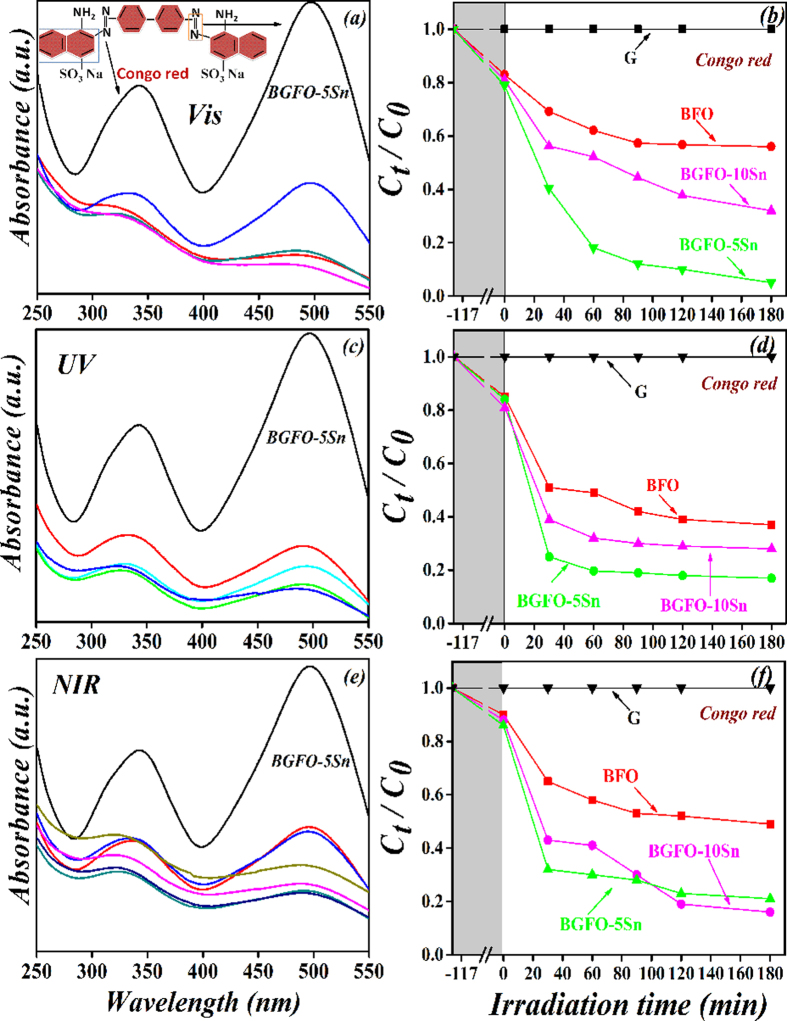
Absorption spectra and photocatalytic degradation efficiencies of Congo red solution in the presence of BGFO-5Sn powder under irradiation of (**a**,**b**) visible (420 nm < λ < 780 nm), (**c**,**d**) UV (λ = 365±5 nm), and (**e**,**f**) NIR (800 nm < λ < 1100 nm) lights, where G represents the degradation of Congo red without light and the shaded area shows degradation of Congo red with the catalyst in the dark for 2 h.

Here, *C*_*o*_ represents the initial concentration of organic dye and *C* represents the ultimate concentration of organic dye degraded after the specified time interval *t*^54^. The absorbance spectra of CR solution was analyzed by using a UV-*vis*-NIR spectrophotometer after regular intervals of time by comparing it with the maximum band absorption at 496 nm. The comparison for photocatalytic degradation efficiency of CR with pure and co-doped BFO nanoparticles under UV-*vis*-NIR light is shown in Table 1. The pure BFO is less active for CR under UV-*vis*-NIR light, while co-doping of Gd^3+^ and Sn^4+^ into BFO significantly enhances the photocatalytic behavior. Generally, the doping of Sn^4+^ into BGFO up to 5% exhibits the maximum efficiency under UV-*vis*-NIR light as shown in Fig. 5, and further increment of Sn^4+^ into BGFO may reduce its photocatalytic activity as shown in the Supplementary information (Fig. S2).

**Figure 6:**
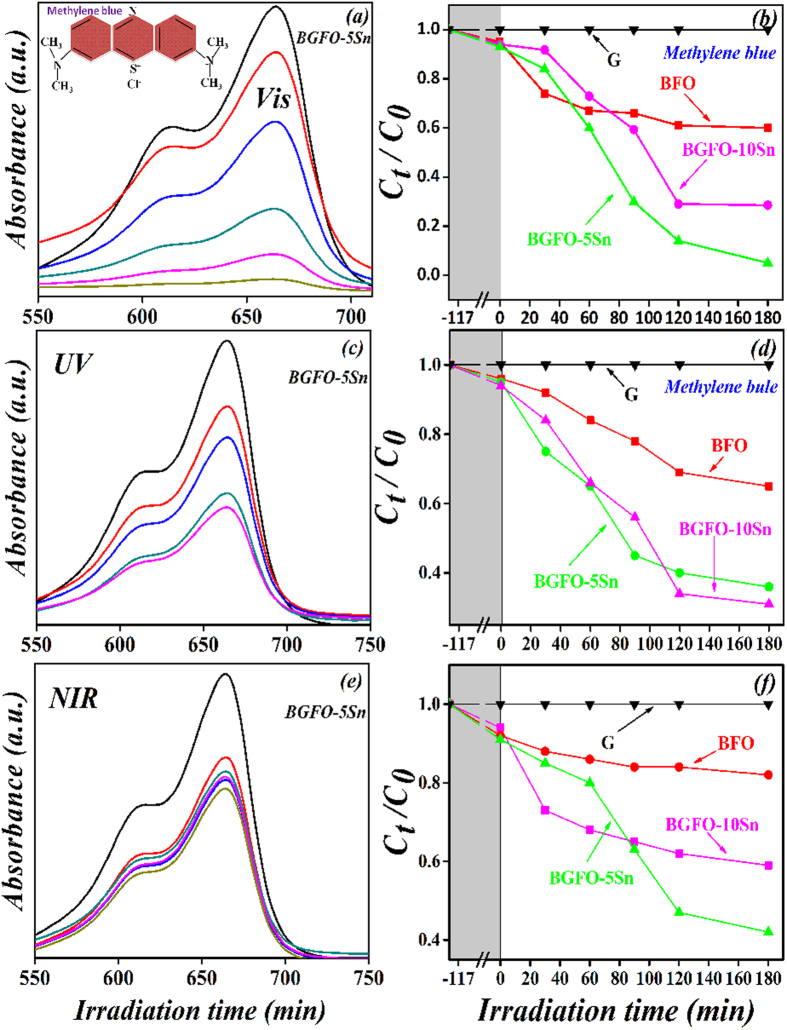
Absorption spectra and photocatalytic degradation efficiencies of Methylene blue solution in the presence of BGFO-5Sn powder under irradiation of (**a**,**b**) visible (420 nm < λ < 780 nm), (**c**,**d**) UV (λ = 365±5 nm), and (**e**,**f**) NIR (800nm < λ < 1100 nm) lights, where G represents the degradation of Methylene blue without light and the shaded area shows degradation of Methylene blue with the catalyst in the dark for 2 h.

Similarly, the absorbance spectrum of MB organic solution was recorded by comparing it with the maximum band absorption at a wavelength of 664 nm. The comparison for photocatalytic degradation efficiency of MB with pure and co-doped BFO nanoparticles under UV-*vis*-NIR light is shown in Table 1. It also shows that the pure BFO is less active for MB degradation under UV-*vis*-NIR light, while co-doping of Gd^3+^ and Sn^4+^ into BFO increases its photocatalytic behavior. Also, the BGFO-5Sn generally shows the maximum efficiency under UV-*vis*-NIR light (Fig. 6), and further increment of Sn^4+^ may reduce the photocatalytic activity for MB (Fig. S3).

**Figure 7:**
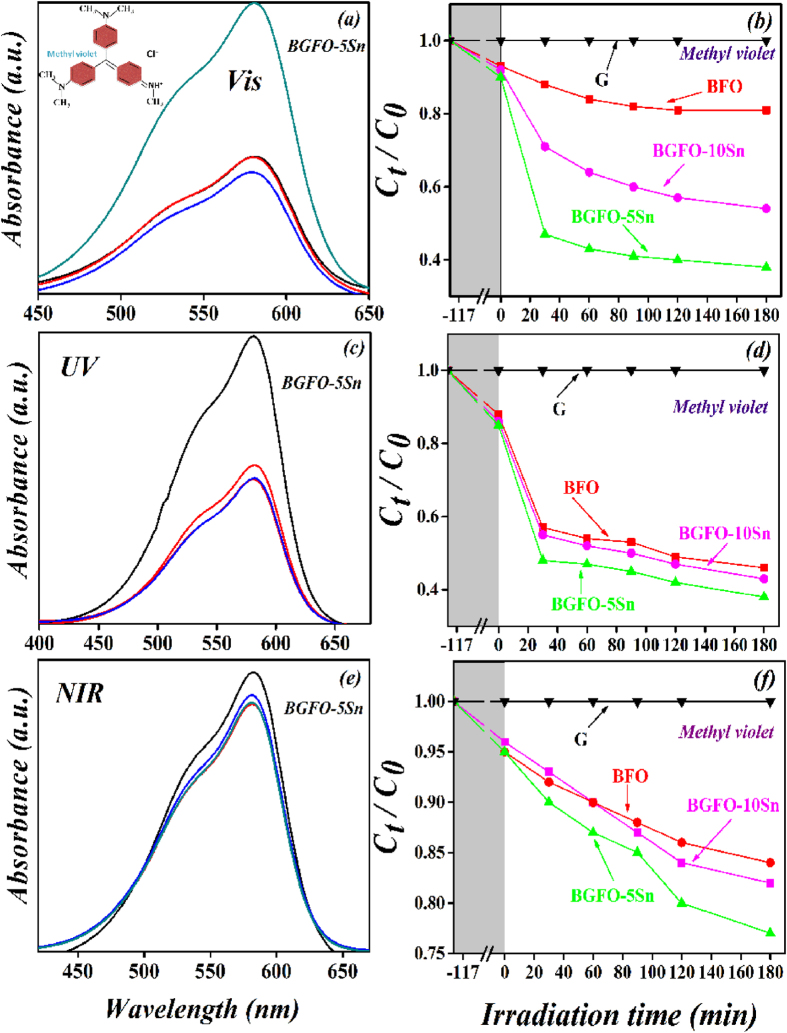
Absorption spectra and photocatalytic degradation efficiencies of Methyl violet solution in the presence of BGFO-5Sn powder under irradiation of (**a**,**b**) visible (420 nm < λ < 780 nm), (**c**,**d**) UV (λ = 365±5nm), and (**e**,**f**) NIR (800nm < λ < 1100 nm) lights, where G represents the degradation of Methyl violet without light and the shaded area shows degradation of Methyl violet with the catalyst in the dark for 2 h.

**Figure 8:**
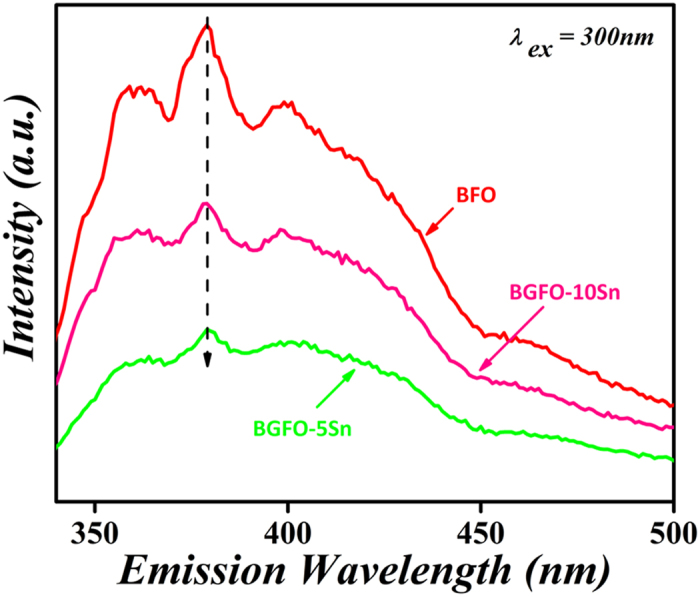
Photoluminescence emission spectra of BFO and BGFSO.

At last, the absorbance spectrum of MV organic solution was recorded by comparing it with the maximum band absorption at a wavelength of 582 nm. The comparison for photocatalytic degradation efficiency of MV with pure and co-doped BFO nanoparticles is shown in Table 1. Pure BFO is less active for MV degradation under UV-*vis*-NIR light, whereas the co-doping of Gd^3+^ and Sn^4+^ increases the photocatalytic efficiency. The BGFO-5Sn always shows the greatest efficiency under UV-*vis*-NIR light (Fig. 7). Further increment of Sn^4+^ into BGFO reduces its photocatalytic activity for MV (Fig. S4). The less degradation of MV dye is due to the difficulty in the reaction of OH radicals with the photocatalyst because of active sites deactivated by strong bye products formation. It is clear that doping of Gd^3+^ and Sn^4+^ into BFO influences well the degradation rate. Photocatalytic activity can also be affected by competition between the charge separation and recombination processes, and the photoluminescence emission spectra have been widely used to estimate the rate of charge recombination^55^,^56^. Figure 8 shows the photoluminescence emission spectra of Sn^4+^ doped BGFO nanoparticles and pure BFO nanoparticles. The BGFO-5Sn nanoparticles show the lowest photoluminescence emission intensity. According to the previous studies, the lower the photoluminescence emission intensity, the lower is the recombination rate of the photo-generated electron-hole pairs and the higher is the photo-activity of photo-catalyst^57^,^58^. It should be noted that the BGFO-10Sn sample shows a higher photoluminescence emission intensity than the BGFO-5Sn sample. This could be attributed to the fact that the extra amount of Sn^4+^ dopants in the BGFO-10Sn sample would possibly produce more surface defects that capture the photo-induced electrons to further create excitons, which hence leads to the enhanced photoluminescence emission intensity. It is most likely that the co-doped Gd^3+^ and Sn^4+^ play a key role in hampering the recombination of electron-hole pairs and as a result, the photo-activity of BGFO-5Sn nanoparticles improves significantly. Many factors such as particles size, crystallinity, surface area, polarization conductivity, and band-gap may affect photocatalytic degradation. In our system, the reduced particle size (60 nm to 20 nm), increased surface area (3.3 m^2^/g to 15 m^2^/g), and suppression of the recombination rate of electron and holes can be the major factors for the enhanced photocatalytic activity of Gd^3+^ and Sn^4+^ co-doped BFO nanoparticles system.

### Photocatalytic mechanism

The photocatalytic mechanism usually involves three steps: (i) the absorption of photons with an energy greater than the band-gap of a photocatalyst, (ii) the production, separation, transfer or recombination of photo-generated e^−^-h^+^ pairs, and (iii) the oxidation-reduction reactions on the photocatalyst surface. The BGFO-5Sn sample shows much higher photocatalytic activities than the pristine BFO. This could be due to the following three aspects: (1) the expansion of excitation wavelength, (2) the decrease of e^−^-h^+^ pair recombination, and (3) the promotion of surface redox reactions. It can be seen from the photoluminescence spectra that there is an optimum doping concentration of rare earth ions Gd^3+^ and Sn^4+^ in BFO for the most efficient separation and migration of photo-generated e^−^ and h^+^, which could be discussed in terms of the space-charge layer thickness^59^. It is known that the value of the space charge layer thickness for the effective separation of photo-generated charge carriers must not be lower than a critical value^60^ and the thickness of the space charge layer can be changed by dopant concentration under the following equation^61^,


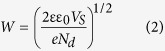


where *W* is the thickness of the space charge layer, *ε* and *ε*_*0*_ are the static dielectric constants of the semiconductor and the vacuum, respectively, *N*_*d*_ is the number of dopant atoms, *V*_*s*_ is the surface potential, and *e*^*−*^ is the electronic charge. Thus, by increasing the number of dopant atoms, the thickness of space charge layer would decrease^62^. Therefore, there should be a specific rare earth dopant concentration that makes the thickness of the space charge layer considerably equal to the light penetration depth^63^.

As the dopant concentration of Gd^3+^ and Sn^4+^ increases towards the optimum value, the surface barrier becomes higher, which hence makes the space charge region narrower and results in a more efficient separation of the e^−^-h^+^ pairs within the region. When the Gd^3+^ and Sn^4+^ dopant concentration is above its optimum value, the space-charge region becomes much smaller, and the light penetration depth into the photocatalyst goes beyond the space-charge layer. Therefore, the recombination of the e^−^-h^+^ pairs becomes easier under these circumstances, making the photocatalytic activities of the photocatalyst decrease. Hence, there is an optimum Gd^3+^ and Sn^4+^ dopant concentration in the co-doped BFO samples for which the photocatalytic activity is the best. A possible mechanism for the improved photocatalytic degradation of Congo red by Gd^3+^ and Sn^4+^ co-doped BFO photocatalyst is proposed as follows. The Gd^3+^ and Sn^4+^ co-doped BFO nanoparticles are excited by visible-light (λ ≥ 420 nm) to produce photo-generated e^−^ and h^+^ (Equation 3). This co-doping into BFO acts as electron trapping sites^64^,^65^ that capture excited electrons (Eq. 4) and make the separation of e^−^-h^+^ pairs possible, therefore supporting the transfer of charges from bulk BFO to the surface of the photocatalyst. Thus, the photo-induced electrons transferred from the Gd^3+^ and Sn^4+^ co-dopants to the photocatalyst surface could capture the adsorbed O_2_ and convert it into 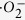 radicals (Eq. 5). The reduced 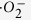 would further contribute towards CR degradation reactions (Eq. 8). Simultaneously, the photo-generated holes after moving towards the surface of the photocatalyst could also react with H_2_O to form ·OH (Eq. 6) for the degradation of CR (Eq. 9) or directly oxidize the CR (Eq. 7). According to the trapping experiments, holes and superoxide radicals are assumed to be the predominant reactive species for CR degradation (Eqs 7 and 8) according to the trapping experiments, while ·OH could also play minor roles in the degradation activity (Eq. 9). Finally, the CR is mineralized into CO_2_, H_2_O, or inorganic ions. The proposed photocatalytic mechanism of Gd^3+^ and Sn^4+^ co-doped BFO for CR degradation is expressed as follows:


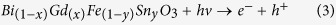



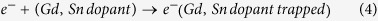



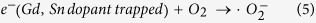



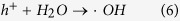



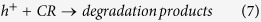



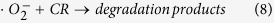



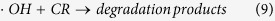


**Figure 9:**
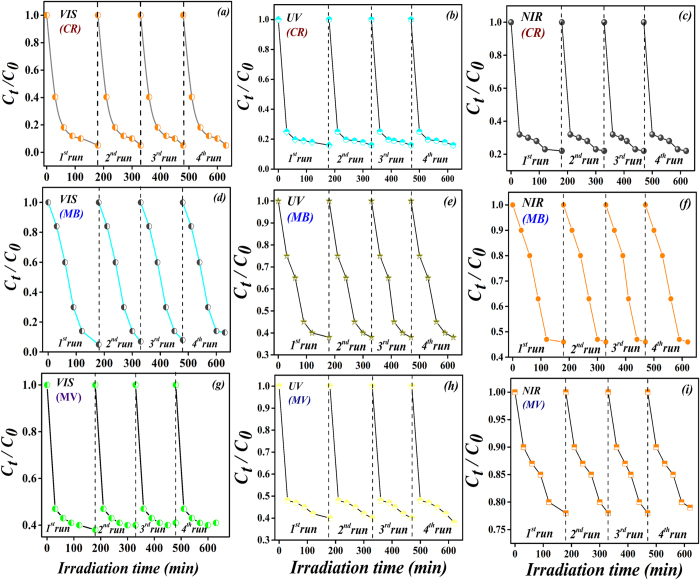
Cycling runs using BGFO-5Sn powder for photo-degradation of Congo red (CR), Methylene blue (MB), and Methyl violet (MV) under irradiation of visible (420 nm < λ < 780 nm), UV (λ = 365±5 nm), and NIR (800 nm < λ < 1100 nm) lights.

In addition, the reusability and stability of a photocatalyst are important factors for practical applications. To estimate the reusability and stability of Gd^3+^ and Sn^4+^ co-doped BFO photocatalyst, the BGFO-5Sn photocatalyst was recycled 4 runs for photodegradation of CR, MB, and MV as shown in Fig. 9. After 4 successive runs with each reaction fixed for 180 min, the degradation efficiency of BGFO-5Sn photocatalyst was mostly maintained, showing a good stability. This result also confirmed that the degradation of CR, MB, and MV by Gd^3+^ and Sn^4+^ co-doped BFO photocatalyst was a photocatalytic reaction rather than a photo-corrosive reaction.

## Conclusions

In summary, we reported the effects of co-doping of Gd^3+^ and Sn^4+^ on the photocatalytic properties of BFO. The BGFO-5Sn nanoparticles possessed a mesoporous nature with a high surface area. The co-doping of Gd^3+^ and Sn^4+^ suppressed the recombination of electron-hole pairs. As a result, the photocatalytic degradation performance of BGFO-5Sn nanoparticles for Congo red, Methylene blue, and Methyl violet was improved significantly in comparison with pure BFO nanoparticles. More importantly, the BGFO-5Sn system acted as a photocatalyst in a broad solar spectrum region from UV to NIR and showed good reusability and stability. Our work proposed new perspectives for efficient photocatalysts that can degrade different organic dyes under irradiation with various light wavelengths and gave guidance for designing more efficient photocatalysts in future.

## Materials and Methods

### Materials

The Bi_1−x_Gd_x_Fe_1−y_Sn_y_O_3_ (as BGFSO) (x = 0.0, 0.01; y = 0.0, 0.05, 0.10) abbreviated as BiFeO_3_ (BFO), Bi_0.90_Gd_0.10_Fe_0.95_Sn_0.05_O_3_ (BGFO–5Sn), and Bi_0.90_Gd_0.10_Fe_0.90_Sn_0.10_O_3_ (BGFO–10Sn) nanoparticles were synthesized by double solvent sol-gel method. The Bi(NO_3_)_3_·5H_2_O (99% pure) and Gd(NO_3_)_3_·6H_2_O (99.9% pure) were mixed stoichiometrically, dissolved in acetic acid [C_2_H_4_O_2_] and ethylene glycol [C_2_H_6_O_2_], and stirred for 90 min at room temperature (RT). Fe(NO_3_)_3_·9H_2_O (98.5% pure) and Tin (Sn) powders were dissolved in acetic acid with a constant magnetic stirring for 1.5 h. After this, both the solutions were mixed and set to a constant stirring for 3 h. A uniform, reddish brown, and fine precursor solution (0.4 M) was produced. To compensate the bismuth loss during the heating process, solutions were synthesized with 3% excess of bismuth. Ethylene glycol was used as the solvent that maintained electronegativities of iron and bismuth during the chemical reaction. Acetic acid was used as a catalyst that maintained the solution’s concentration and controlled chemical reaction during the synthesis process. The as-prepared solution was dried in an oven at 80 °C for 12 h to get a gel and then calcined in furnace at 600 °C for 3 h. After that, it was crushed to get a fine powder.

### Characterization

The phase constitutions of BGFSO nanoparticles were characterized by X-ray diffractometry (XRD, Rigaku 2500, Japan) with Cu-Ka radiation operating at 40 kV and 20 mA. The scanning electron microscopy (SEM, JSM-6460, Japan) was used to study the morphology of BGFSO nanoparticles. The band-gap and photocatalytic properties of BGFSO nanoparticles were studied by UV-vis diffused reflectance spectra *via* UV- *vis* spectrophotometry (Hitachi UV-3310, Japan) with an integration sphere. The porosity and Brunauer-Emmett-Teller (BET) surface area of the samples were obtained from N_2_ sorption/desorption isotherms at 77 K by Quadrasorb-SI v. 5.06 (Quantachrome Instruments Corporation, USA). The photoluminescence spectra were measured on Horiba Scientific Fluoromax-4 spectrofluorometer using 300 nm excitation wavelengths.

## Additional Information

**How to cite this article**: Irfan, S. *et al*. The Gadolinium (Gd^3+^) and Tin (Sn^4+^) Co-doped BiFeO_3_ Nanoparticles as New Solar Light Active Photocatalyst. *Sci. Rep.* **7**, 42493; doi: 10.1038/srep42493 (2017).

**Publisher's note:** Springer Nature remains neutral with regard to jurisdictional claims in published maps and institutional affiliations.

## Supplementary Material

Supplementary Information

## Figures and Tables

**Table 1:** Comparison for photocatalytic degradation efficiencies of CR, MB, and MV organic dyes in the presence of pure and Gd^3+^ and Sn^4+^ co-doped BFO nanoparticles under irradiation with different wave lengths.

Samples	Degradation Under			Degradation Under			Degradation Under		
	Vis			UV			NIR		
	CR	MB	MV	CR	MB	MV	CR	MB	MV
BFO	44%	40%	19%	63%	35%	54%	51%	18%	16%
BGFO-5Sn	95%	95%	62%	83%	64%	62%	79%	58%	23%
BGFO-10Sn	68%	72%	46%	72%	69%	57%	84%	41%	18%
