# Supplementary material for: The Gadolinium (Gd3+) and Tin (Sn4+) Co-doped BiFeO3 Nanoparticles as New Solar Light Active Photocatalyst
Source: Sci Rep. 2017 Feb 14;7:42493. doi: 10.1038/srep42493 (PMC5307368; doi:10.1038/srep42493)
Supplement: Supplementary Information [file srep42493-s1.pdf]

Supplementary Information

The Gadolinium (Gd^{3+}) and Tin (Sn^{4+}) co-doped $BiFeO_3$ nanoparticles as new solar light active photocatalyst

Syed Irfan [1], Syed Rizwan [2,*], Yang Shen [1], Liangliang Li [1,*], Asfandiyar [1], Sajid Butt [3], Ce-Wen Nan [1]

[1]State Key Laboratory of New Ceramics and Fine Processing, School of Materials Science and Engineering, Tsinghua University, Beijing 100084, China

[2]Department of Physics, School of Natural Sciences (SNS), National University of Science and Technology (NUST), Islamabad 44000, Pakistan

[3]Department of Materials Science and Engineering, Institute of Space Technology, Islamabad, 44000, Pakistan

Correspondence and requests for materials should be addressed to L. Li (E-mail: liliangliang@mail.tsinghua.edu.cn; Fax: +86-10-62771160; Tel: +86-10-62797162) or S. Rizwan (E-mail: syedrizwanh83@gmail.com; Tel: +92-51-90855599).

Table S1. Comparison of BET analysis of pure and Gd^{3+} and Sn^{4+} co-doped BFO nanoparticles.

Samples	Surface area<br>m^2/g	Pore size<br>nm	Pore volume<br>cc/g
BFO	3.3	1.7	0.02
BGFO-5Sn	15	2.2	0.06
BGFO-10Sn	12	2.2	0.07

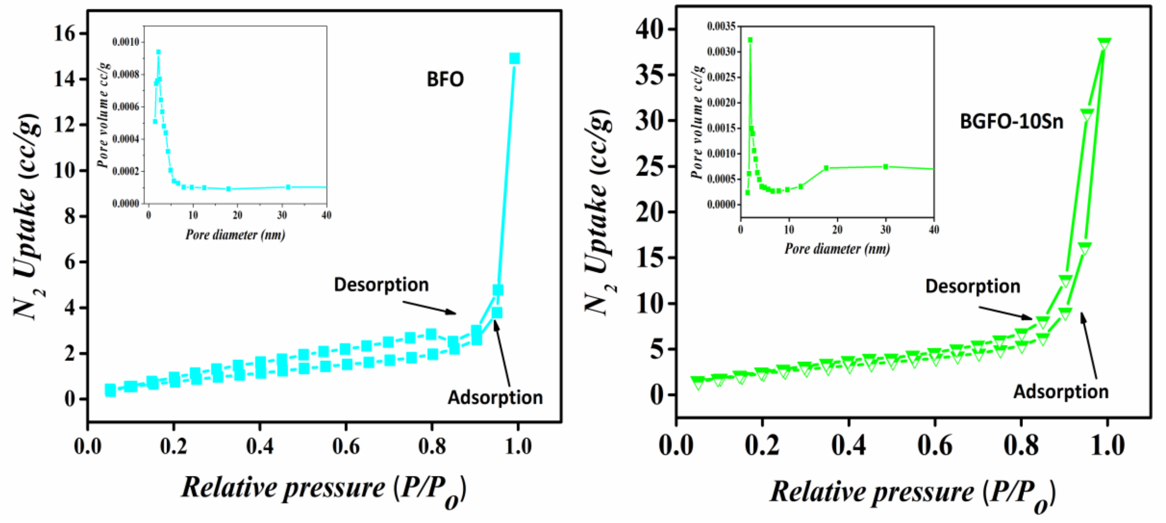

Figure S1. N_2 gas isotherms measured at 77K for pure BFO and BGFO-10Sn. The insets are the differential pore size distribution curves from BJH method.

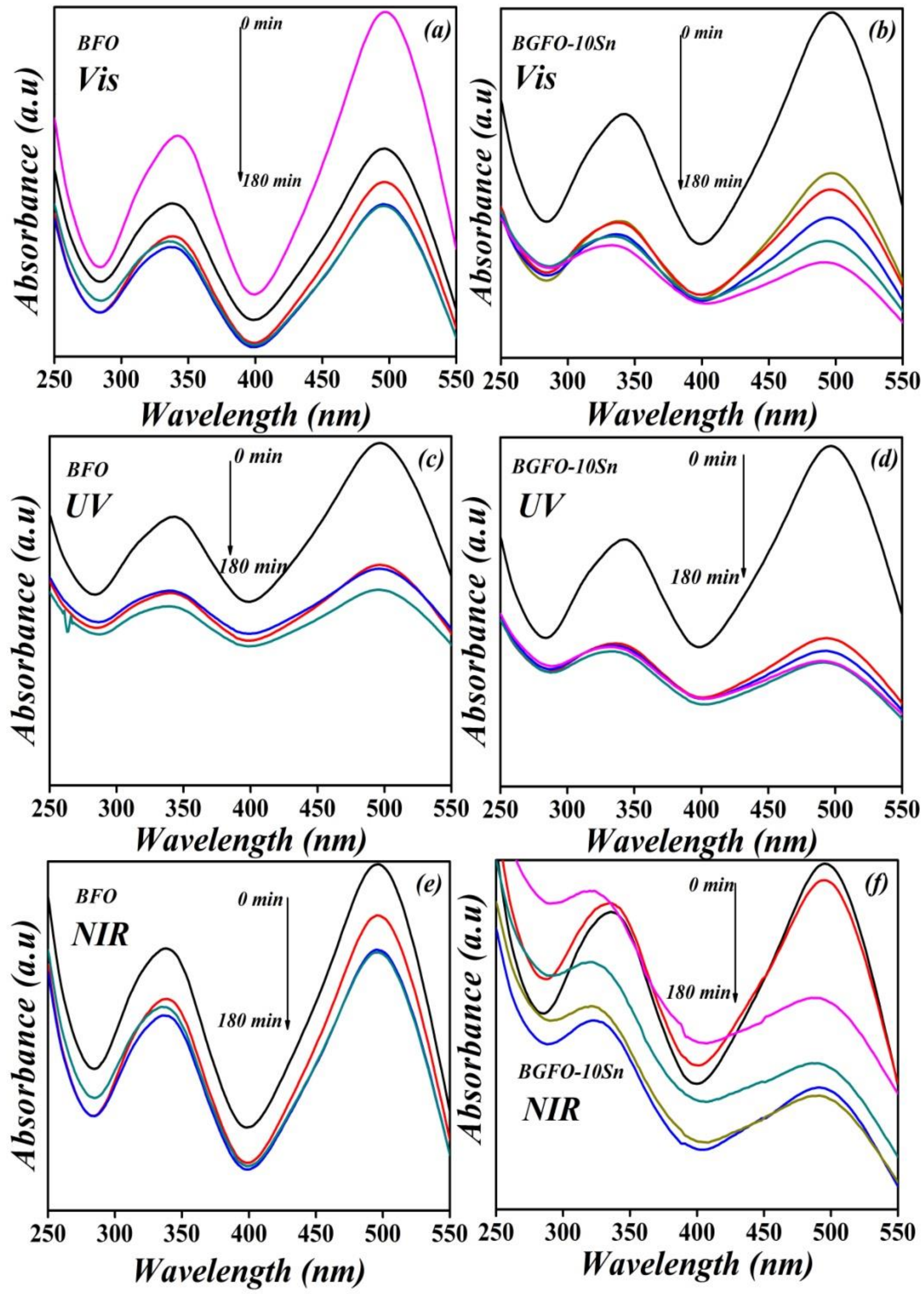

Figure S2. Absorption spectra of CR solution degraded in the presence of (a, c & e) BFO and (b, d & f) BGFO-10Sn under visible, UV, and NIR irradiation.

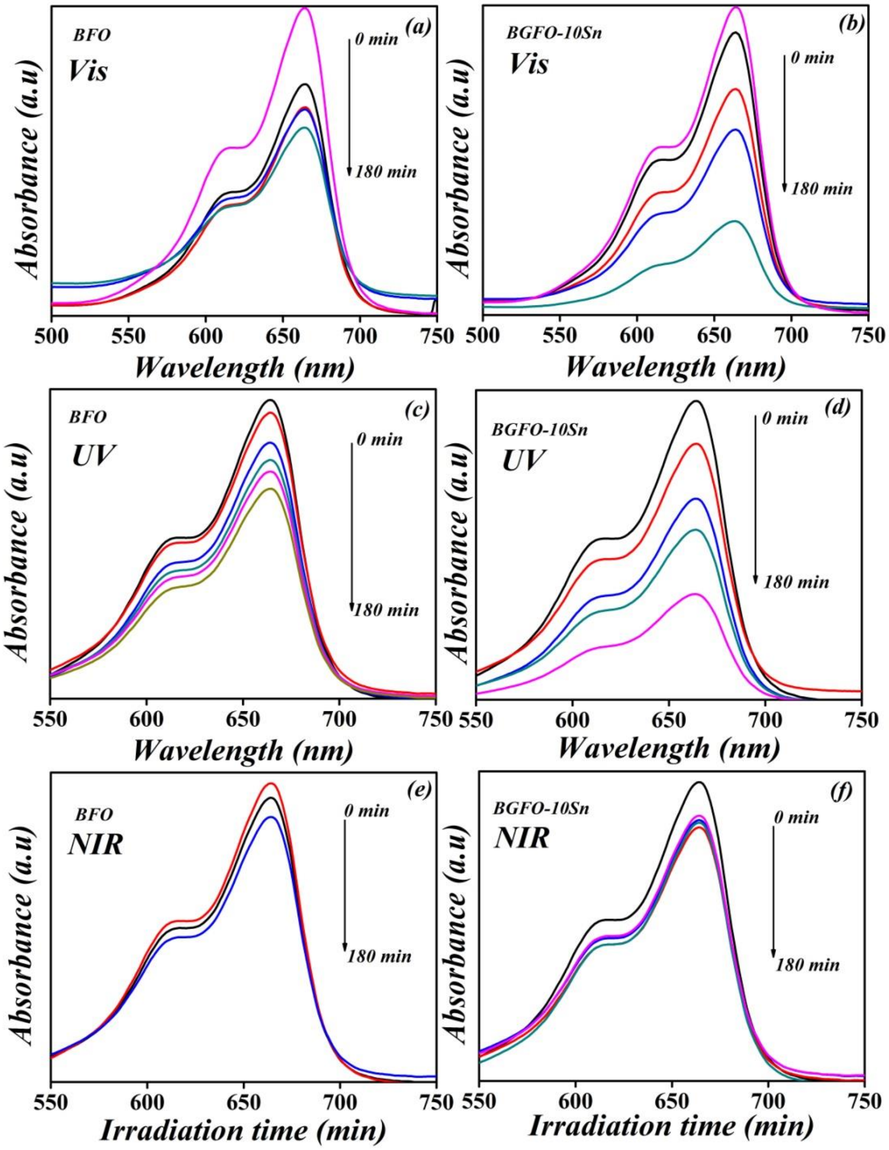

Figure S3. Absorption spectra of MB solution degraded in the presence of (a, c & e) BFO and (b, d & f) BGFO-10Sn under visible, UV, and NIR irradiation.

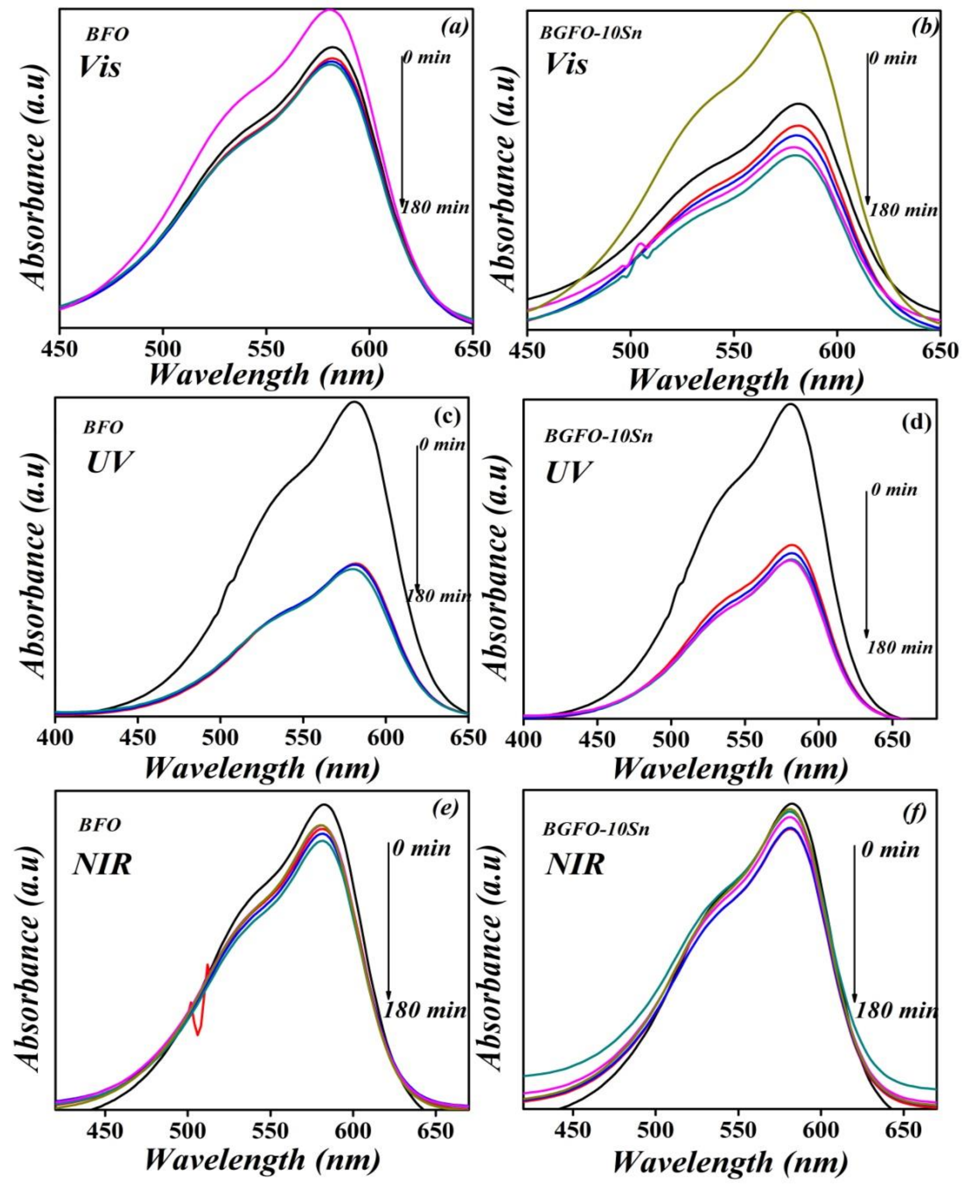

Figure S4. Absorption spectra of MV solution degraded in the presence of (a, c & e) BFO and (b, d & f) BGFO-10Sn under visible, UV, and NIR irradiation.
